# Long-term lithium therapy and risk of chronic kidney disease, hyperparathyroidism and hypercalcemia: a cohort study

**DOI:** 10.1186/s40345-023-00286-8

**Published:** 2023-01-29

**Authors:** Elise Boivin, Brendan Le Daré, Romain Bellay, Cécile Vigneau, Marion Mercerolle, Astrid Bacle

**Affiliations:** 1 Pôle Pharmacie, Service Hospitalo-Universitaire de Pharmacie, CHU Rennes, 35000 Rennes, France; 2 Institut NuMeCan (Nutrition, Metabolismes et Cancer), Réseau PREVITOX, INSERM, INRAE, Université de Rennes 1, Rennes, France; 3 Service Pharmacie, Centre Hospitalier Guillaume Regnier, Rennes, France; 4 Service de Néphrologie, Centre Hospitalier Universitaire Pontchaillou, Rennes, France; 5 Univ Rennes, CHU Rennes, INSERM, EHESP, Irset-UMR_S 1085, 35000 Rennes, France

**Keywords:** Lithium, Bipolar disorder, Nephrotoxicity, Hyperparathyroidism, Hypercalcemia

## Abstract

**Background:**

Lithium is well recognized as the first-line maintenance treatment for bipolar disorder (BD). However, besides therapeutic benefits attributed to lithium therapy, the associated side effects including endocrinological and renal disorders constitute important parameters in prescribing patterns and patient adherence. The objectives of this study is to (i) determine whether long-term lithium therapy is associated with a decrease in renal function, hyperparathyroidism and hypercalcemia and (ii) identify risk factors for lithium-induced chronic kidney disease (CKD).

**Methods:**

We conducted a single-centered cohort study of adult patients (≥ 18 years) treated with lithium, who were enrolled at Rennes University Hospital in France between January 1, 2018 and June 1, 2020. Required data were collected from the patient’s medical records: demographics characteristics (age, sex, body mass index), biologic parameters (GFR, lithium blood level, PTH and calcium), medical comorbidities (hypertension and diabetes), lithium treatment duration and dosage, and length of hospitalization.

**Results:**

A total of 248 patients were included (mean age: 60.2 ± 16.5 years). Duration of lithium treatment correlated with (i) deterioration of renal function estimated at − 2.9 mL/min/year (p < 0.0001) and (ii) the development of hyperparathyroidism (p < 0.01) and hypercalcemia (p < 0.01). We also noted that patients with lithium blood level > 0.8 mEq/mL had significantly lower GFR than patients with lithium blood level < 0.8 mEq/mL (61.8 mL/min versus 77.6 mL/min, respectively, p = 0.0134). Neither diabetes mellitus nor hypertension was associated with more rapid deterioration of renal function.

**Conclusion:**

This study suggests that the duration of lithium treatment contribute to the deterioration of renal function, raising the question of reducing dosages in patients with a GFR < 60 mL/min. Overdoses has been identified as a risk factor for CKD, emphasizing the importance of regular re-evaluation of the lithium dose regimen. Also, long-term lithium therapy was associated with hyperparathyroidism and hypercalcemia. Particular vigilance is required on these points in order to limit the occurrence of endocrinological and renal lithium adverse effects.

## Introduction

Lithium is well recognized as the first-line maintenance treatment for bipolar disorder (BD) in all international clinical practice guidelines since it is effective in preventing relapses of mood episodes and in reducing risk of suicide (Geddes and Miklowitz 2013), (Goodwin et al. 2016; Yatham et al. 2018). Others therapeutic indications of lithium therapy include schizophrenia (Leucht et al. 2015), major depression (Vázquez et al. 2021), alcoholism and cluster headaches (Timmer and Sands 1999). Besides therapeutic benefits attributed to lithium therapy, the associated side effects constitute important parameters in prescribing patterns and patient adherence.

Among the endocrinological effects, lithium-induced hypercalcemia and hyperparathyroidism are well known in the literature and appear to result from both acute and chronic effects. The acute, potentially reversible effects are related to the action of lithium on the calcium-sensing receptor and glycogen synthase kinase 3 pathway, resulting in a biochemical picture similar to that seen in familial hypocalciuric hypercalcemia (Ballehaninna et al. 2011; Mifsud et al. 2020; Szalat et al. 2009). Chronic effects are thought to be related to permanent changes in the parathyroid glands, either by unmasking hyperparathyroidism in patients with subclinical parathyroid adenoma or by triggering multiglandular hyperparathyroidism (McHenry and Lee 1996; Mifsud et al. 2020; Nordenström et al. 1992).

Among nephrotoxic effect, lithium is well known to cause nephrogenic diabetes insipidus (NDI) with an incidence that might be as high as 85% (Bockenhauer and Bichet 2015; Hetmar et al. 1986; Rej et al. 2012). Discontinuation of lithium therapy may resolve the symptoms of NDI, but this approach is not a reasonable treatment option in most cases, as the beneficial effects of lithium on psychiatric disorders overcome the negative impact of polyuric complications on quality of life. Even if the exact mechanism of this lithium toxicity is not known, robust data suggest that lithium exerts its effects after entering the principal cell through epithelial sodium channels (ENaCs), which have high permeability for lithium (Kortenoeven and Fenton 2014). This mechanism notably allows for the proposal of ENaC blockers such as amiloride, to increase urine osmolality and improve polyuria in lithium-induced NDI (Batlle et al. 1985; Bedford et al. 2008; Kortenoeven et al. 2009).

Beside lithium-induced NDI, its ability to cause chronic kidney disease (CKD) is a much more debated issue in the literature, with various studies suggesting both a positive and negative relationship (Azab et al. 2015; Clos et al. 2015; Gitlin 2016) (Gupta and Khastgir 2017). Despite these conflicting data, the predominant view seems to be that lithium has the capacity to cause chronic tubulointerstitial nephritis leading to progressive CKD over several years and end stage renal disease (ESRD) in about 1.5% of long-term lithium users (Fogo et al. 2017). To go further, the definition of risk factors for the development of CKD on lithium remains to date very poorly documented. Answering this question is all the more difficult because the progression of CKD on lithium may be irreversible. This makes it particularly difficult to observe an improvement in renal function when a suspected risk factor is removed. In contrast to NDIs, the question of the benefit-risk balance in the face of a patient with CKD with a high risk of relapse of BD if treatment is stopped is thus more challenging and currently constitutes an obstacle to the development of detailed recommendations on the subject (Ng et al. 2009).

Hence, the objectives of this study is to (i) determine whether long-term lithium therapy is associated with a decrease in renal function and hyperparathyroidism (ii) identify risk factors for lithium-induced nephrotoxicity.

## Materials and methods

### Study design and setting

This study was designed as a single-centered. We conducted a cohort study of adult patients (≥ 18 years) on lithium, who were hospitalized for any cause more than 24 h in Rennes University Hospital in France between January 1, 2018 and June 1, 2020. No specific ethical approval was sought. All information used within the study was anonymized and not traceable to a single individual.

### Data sources

We collected the required data from the patient’s medical records: demographics characteristics [age, sex, body mass index (BMI)], biologic parameters (glomerular filtration rate (GFR), lithium blood level, parathormone (PTH) and calcium), medical comorbidities (hypertension and diabetes), lithium treatment duration and dosage. Only biological parameters at entry were considered. For lithium blood level assessment, blood samples from patients on once-daily lithium therapy were collected 24 h after the last dose, while blood samples from patients on twice-daily lithium therapy were collected 12 h after the last dose. GFR were estimated by CKD-EPI equations. Common nephrotoxic drugs at admission and discharge considered in this study (and listed according to the literature) are displayed in Table 1 (Kwiatkowska et al. 2021; Mody et al. 2020; Perazella 2018; Perazella and Rosner 2022).

**Table 1:** Most commonly found nephrotoxic drugs

Drug class	Descriptions
Antibiotics	Aminosides, beta-lactams (penicillins and cephalosporins), fluoroquinolones, sulfonamides, rifampicin, vancomycin
Antifungal agents	Amphotericin B
Anti-rheumatic	NSAIDs, allopurinol, biphosphonates
Antivirals	Aciclovir, adefovir, cidofovir, foscarnet, ganciclovir, tenofovir
Immunosuppressants	Methotrexate, tacrolimus, cyclosporine, interferon, high dose interleukin 2, immunoglobulins
Cardiology	Angiotensin-converting enzyme (ACE) inhibitors, Angiotensin II receptor blockers (ARB), aliskiren, dextran, loop diuretics, thiazide diuretics, methyldopa
Gastroenterology	PPIs, H2 blockers, mesalazine
Haemostasis	Clopidogrel, ticlopidine
Hormones modulators	propylthiouracil
Nervous system	Carbamazepine, phenobarbital, phenytoin, valproic acid
Radio Constrat Agents	Iodated radiocontrast agents

### Statistical analysis

The significance of intergroup differences [expressed as the mean ± standard error of the mean (SEM)] was determined using an unpaired t-test. A chi-squared test was used to compare percentages. Linear regression analysis was used to assess the relationship between biological variables (GFR, lithium blood level, PTH and calcium) and time of lithium exposure. Multiple regression analysis were used to adjust GFR for age, sex, number of nephrotoxic medications, hypertension, diabetes and of time of lithium exposure. All analyses were performed using Prism software (version 8.0, GraphPad Software, La Jolla, CA, USA). The threshold for statistical significance was set to *p* < 0.05 in all cases.

## Results

### Sample description

A total of 248 patients were included. Table 2 summarizes the clinical characteristics and demographics of patients. In the cohort, 59% (n = 146) of patients were women, mean age 60.2 ± 16.5 years. The majority of patients, 71% (n = 176) were on lithium for bipolarity. Co-occurring hypertension and diabetes mellitus were found in 80 patients (32%) and 34 patients (14%), respectively. Twenty-one patients (8.5%) presented both hypertension and diabetes mellitus.

**Table 2:** Characteristics and demographics of patients

	Total (n = 248)
Ages, mean ± SD in years	60.2 ( ± 16.5)
Female, n (%)	146 (59)
BMI mean ( ± SD)	27.2 ( ± 6.7)
Etiology, n (%)	
Bipolarity	176 (71.0)
Depressive syndrome	32 (12.9)
Schizophrenia	21 (8.5)
Mixed anxiety-depressive disorder	11 (4.4)
Unknown	3 (1.2)
Others	5 (2.0)
Duration on lithium in years (mean ± SD) (n = 236)	10.6 ( ± 10.9)
Number of patients with lithium blood level	115 (46.3)
Lithium blood level (mEq/L)	0.9 ( ± 0.8)
< 0.5 (n = 36)	0.3 ( ± 0.1)
[0,5–0,8] (n = 36)	0.6 ( ± 0.1)
]0,8–1,20] (n = 25)	1.0 ( ± 0.1)
> 1,2 (n = 18)	2.3 ( ± 1.0)
Extended-release formulation, n (%)	199 (80.2)
Diabetes Mellitus, n (%)	34 (14)
Hypertension, n (%)	80 (32)
GFR in ml/min (mean ± SD) (n = 243)	77.5 ( ± 31.9)
Stage 1 ≥ 90 (n = 102)	106 ( ± 11.4)
Stage 2 60–89 (n = 71)	76.8 ( ± 8.8)
Stage 3 30–59 (n = 47)	45.4 ( ± 9.1)
Stage 4 15–29 (n = 15)	24.3 ( ± 4.1)
Stage 5 < 15 (n = 8)	9.7 ( ± 2.9)

Mean lithium therapy duration was 10.6 ± 10.9 years corresponding to an estimated cumulative lithium dose of 2221 ± 2570 g per patient. The mean CKD-EPI was 77.5 ± 31.9 mL/min per 1.73 m^2^ and 70 patients (28.8%) developed CKD stage 3 or more severe (CDK < 60 mL/min/1.73 m^2^). Eight patients reached ESRD at median age of 59 (± 11.3) years.

Mean lithium serum levels was 0.9 ± 0.8 mEq/L. Slightly more than 31% of the patients had a lithium blood level within the norms (i.e. between 0.5 and 0.8 mEq/L) while 37% were overdosed and 31% underdosed. More than half of patients [n = 133 (53.6%)] had no lithium blood level checked during their hospitalization. Regarding the distribution of the lithium dose, more than 86% of psychiatrists prescribe it in a single daily dose, 12% in 2 daily doses and only 2% in 3 daily doses. Lastly, the median age of the extended-release lithium group was significantly lower than the immediate-release lithium group (60 years old versus 66 years old, respectively, p = 0.0068).

### Co-medication

Table 3 reports the nephrotoxic drugs most frequently found on prescriptions at admission and discharge. The number of nephrotoxic drugs per patient at admission tended to be higher in the GFR > 60 ml/min group compared to the < 60 ml/min group although not significant (0.45 ± 0.04 versus 0.37 ± 0.01, respectively).

**Table 3:** Nephrotoxic drugs in order of frequency

Pharmacologic class	At admission (n, %)	Discharge (n, %)
PPI	66 (19.0)	52 (18.4)
ACE inhibitors	45 (13.0)	18 (6.0)
Valproic acid	32 (9.0)	19 (6.7)
Loop diuretics	23 (7.0)	18 (6.4)
Angiotensin II receptor blocker	18 (5.0)	11(3.9)
Thiazide diuretics	16 (5.0)	7 (2.5)
Beta-lactams	12 (3.0)	27 (7.7)
Non-steroidal anti-inflammatory drugs	4 (1.2)	6 (2.1)

Interestingly, decreased in renal function was non-significantly associated with a decrease in the average number of nephrotoxic drugs per patient (Table 4).

**Table 4:** Number of nephrotoxic drugs per patient according to CKD-EPI.

CKD-EPI (mL/min)	Number of patients	Nephrotoxic drugs per patient (mean ± SEM)	p-value (compared to CDK-EPI ≥ 90 group)
≥ 90	102	0.47 ± 0.52	NA
60–89	71	0.44 ± 0.50	0.6685
30–59	47	0,45 ± 0.62	0.8078
15–29	15	0,2 ± 0.41	0.0891
< 15	8	0.25 ± 0.46	0.3057

*PPI* proton pump inhibitors, *ACE* angiotensin-converting enzyme

### Relation between lithium therapy and renal functional impairment

**Fig. 1:**
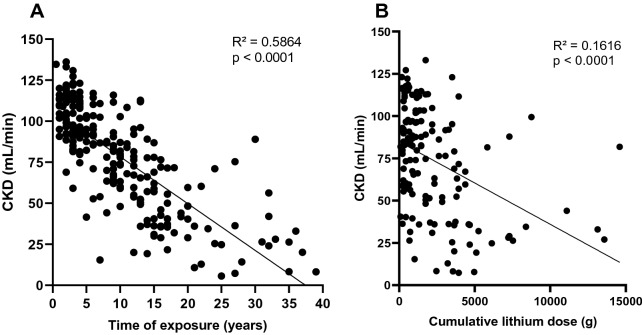
Linear regression analysis between glomerular filtration rate estimated by CKD equation and time of lithium exposure **A** and cumulative lithium dose **B**

Estimated creatinine clearance inversely correlated with the length of lithium therapy (Fig. 1A) (p < 0.0001) and estimated cumulative dose of lithium (Fig. 1B) (p < 0.0001). Deterioration of renal function was estimated at − 2.9 ml/min/year and median time to develop CKD Stage 3 from starting lithium therapy was 15.2 ± 9.6 years. Lastly, we noted that patients with lithium blood level > 0.8 mEq/ml had significantly lower renal function than patients with lithium blood level < 0.8 mEq/ml (61.8 ml/min versus 77.6 ml/min, respectively, p = 0.0134).

### Biological parameter

**Fig. 2:**
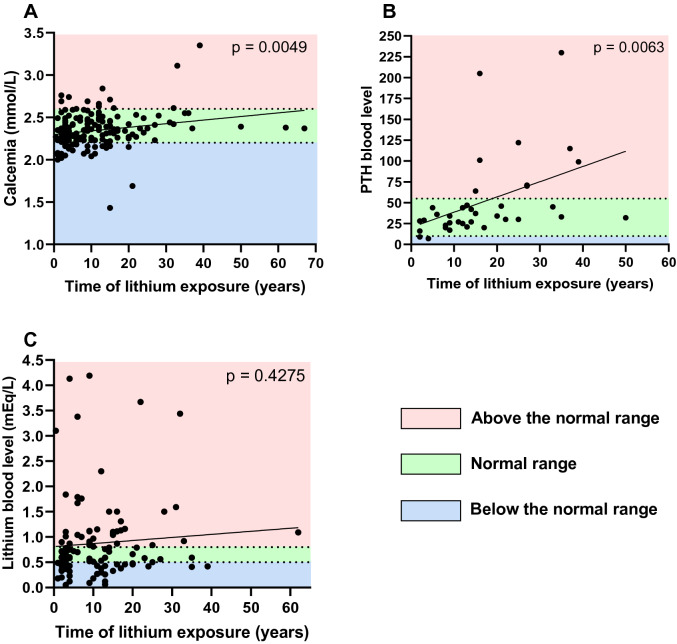
Linear regression analysis between biological variables. **A** Plasma calcium concentration, **B** parathormone (PTH) blood level and **C** lithium blood level according to time of lithium exposure in years

A serum calcium concentration was determinated in 170 patients (2.34 ± 0.21 mmol/L) and 30 (17.6%) patients had hypercalcemia (> 2.5 mmol/L). Parathyroid hormone (PTH) level was measured in 37 patients (51.5 ± 49.0 pg/mL). We found correlation between the number of years on lithium therapy and the increase in calcium (p = 0. 0049) and PTH (p = 0.0063) (Fig. 2A–B). A non-significant trend of increasing blood lithium levels as a function of treatment time was observed (Fig. 2C).

### Co-morbidities

Using multiple regression analysis we investigated whether patient co-morbidities and characteristics (namely age, sex, diabetes mellitus, hypertension, time of lithium exposure and number of nephrotoxic medications) were associated with renal function impairment. We found that age and time of exposure to lithium (but not diabetes mellitus or hypertension) emerged as risk factors for decreased renal function (p < 0.0001) (Table 5).

**Table 5:**
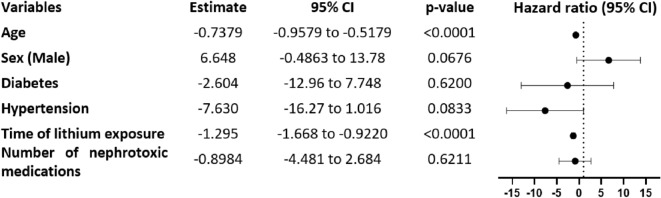
Hazard Ratio for decreased renal function in patients taking lithium, by age, male sex, diabetes mellitus, hypertension, time of lithium exposure and number of nephrotoxic medications

*GFR* glomerular filtration rate, *CI* confidence interval

## Discussion

In this study, we showed that (i) long-term lithium therapy is associated with a decrease in renal function, hyperparathyroidism and hypercalcemia and that (ii) the risk factors identified for renal toxicity should lead to close monitoring of renal function in these patients.

First, the decrease in renal function under lithium therapy was estimated at − 2.9 mL/min/year, which is consistent with previous studies among which this effect was estimated at − 2.3 mL/min/year (Presne et al. 2003; Shine et al. 2015). Multiple regression analysis revealed that time of lithium exposure was a risk factor for decreased renal function after adjusting for age, sex, diabetes, hypertension, and number of nephrotoxic medications (Table 5). The deterioration of renal function is thus 2 to 3 times more rapid compared to the general population where mean annual GFR loss is estimated at approximately 1 mL/min/1.73 m^2^ (Glassock and Rule 2016; Nankivell 2001; Schmitt and Melk 2017). Also, the median time to develop chronic kidney disease stage 3 (< 60 mL/min) from starting lithium therapy was 15.2 years, compared to 21.7 years in previous study (Pahwa et al. 2021). Going further, we highlighted that patients treated with high doses of lithium for a long period of time were likely to be at greater risk of developing chronic kidney disease, and raising the question of dose reduction in patients with a GFR < 60 mL/min. Since we have no data regarding the evolution of lithium dosages over the lifetime of patients, interpretation of cumulative dose data is particularly difficult. Also, a history of acute lithium intoxication is thought to constitute a risk factor for developing lithium-induced chronic renal failure (Gupta and Khastgir 2017). Because of this renal toxicity, a reassessment of the benefit-risk of lithium treatment may be necessary in some patients, although it may not be possible to discontinue it for psychiatric reasons. According to guidelines, patients with GFR < 60 mL/min/1.73 m^2^ require more intensive monitoring, even if the fibrous renal lesions are sometimes irreversible with progression of the deterioration even with the discontinuation of lithium (Kripalani et al. 2009; Morriss and Benjamin 2008).

Second, from a biochemical point of view, we showed that lithium treatment was associated with an increase in blood PTH and calcium concentrations (Fig. 2A, B). Lithium hyperparathyroidism is associated with increased morbidity such as nephrolithiasis and/or reduced bone mineral density, especially in chronically treated patients (Mifsud et al. 2020). For these reasons, regular monitoring of calcium levels in these patients is of utmost importance, as early recognition of lithium-associated hyperparathyroidism may improve outcomes. Also, the literature reports that hypercalcemia and hyperparathyroidism may occur more frequently in the elderly, prompting psychiatrists to be more vigilant both prior to starting treatment and at least annually thereafter (Lehmann and Lee 2013). In addition to the risk of nephrolithiasis, hypercalcemia can worsen nephrogenic diabetes insipidus, leading to dehydration and thus lithium intoxication, which can then deteriorate renal function (Khairallah et al. 2007).

Third, the increase in lithium blood level was not associated with time of lithium exposure in this study (Fig. 2C), despite the associated lower GFR observed for patients with lithium blood level > 0.8 mEq/ml. These results might suggest regular reassessment of renal function and lithium levels in a majority of patients, leading to subsequent adjustment of lithium dosages as the GFR decrease. As a therapeutic range around 1.2 mEq/mL can be targeted in manic episodes, this probably leads to a slight overestimation of the proportion the overdosed patients. This could also explain why the baseline lithium levels are relatively high.

Surprisingly, the presence of comorbidities (namely diabetes and hypertension) were not associated with a more rapid deterioration of renal function (Table 5). In the literature, Pahwa et al. (2021) reported that diabetes mellitus was associated with more rapid deterioration in function, with a population size equal to that of our population (n = 34) (Pahwa et al. 2021). Because of the trend toward accelerated deterioration of renal function observed in the diabetic and hypertensive patients in our study, it is likely that a larger number of patients included would have made a significant difference.

Lastly, it is known that there are several medications which can interfere both with serum lithium levels and renal function whose use needs to be closely monitored. Amongst these include the use of non-steroidal anti-inflammatory drugs, angiotensin-converting enzyme inhibitors, and thiazide diuretics while loop and potassium-sparing diuretics are generally considered to cause less disruption to serum lithium levels (Ng et al. 2009). In our study, the trend toward lower number of nephrotoxic drugs in patients with a GFR < 60 mL/min compared with patients with a GFR > 60 mL/min might suggest a withdrawal of nephrotoxic drugs by clinicians as the GFR decreases. Therefore, the impact of the presence of nephrotoxic drugs on renal function could not be assessed.

This study has several limitations. First, the limited number of patients may have limited the observation of a risk factor for renal toxicity, especially for comorbidities and comedications and a prospective study would be important to perform in this context. Second, the fact that the sample is that of hospitalized patients might indicate that this is a more ill cohort than an outpatient sample. Third, only a minority of the patients were evaluated regarding PTH, which may suggest confounding by indication, leading to caution in interpreting the data. Four, in the age group of the study population the use of D-vitamin and calcium supplement would be likely, which may have slightly overestimated the increases in calcemia found in this study.

Overall, precautions to be taken in case of lithium treatment include:

- Screening for diabetic insipidus
- Regular monitoring of blood lithium levels (we propose every 3 months for the first year, then every 6 months [except high risk groups such as elderly, taking interacting meds, renal or thyroid impairment, poor symptoms control or adherence, last lithium level over 0.8 mmol/L] as suggested by National Health Service guidelines [Tees Esk and Wear Valley NHS Foundation Trust 2021)].
- Biological monitoring (TSH, PTH, ionogram, creatinine at least once a year), associated with education of the patient regarding co-medications or the procedure to follow in case of signs of overdose or dehydratation.

## Conclusion

Lithium therapy result in increased risk of CKD overtime, raising the question of reducing dosages in patients with a GFR < 60 mL/min. Overdoses has been identified as a risk factor for CKD, emphasizing the importance of regular re-evaluation of the lithium dose regimen. Although difficult to implement, a prospective study would be interesting in order to identify more easily the impact of comorbidities and comedications on the evolution of renal function, which did not emerge in our study.

## Data Availability

All data generated or analysed during this study are included in this published article.
